# Epigenetic age and pregnancy outcomes: GrimAge acceleration is associated with shorter gestational length and lower birthweight

**DOI:** 10.1186/s13148-020-00909-2

**Published:** 2020-08-06

**Authors:** Kharah M. Ross, Judith E. Carroll, Steve Horvath, Calvin J. Hobel, Mary E. Coussons-Read, Christine Dunkel Schetter

**Affiliations:** 1 Centre for Social Sciences, Athabasca University, 1 University Drive, Athabasca, AB T9S 3A3 Canada; 2 Department of Psychology, University of Calgary, Calgary, AB Canada; 3 Cousins Center for Psychoneuroimmunology, David Geffen School of Medicine, Semel Institute for Neuroscience and Human Behavior, Department of Psychiatry and Biobehavioral Sciences, University of California – Los Angeles, Los Angeles, CA USA; 4 Department of Biostatistics, University of California – Los Angeles, Los Angeles, CA USA; 5 Department of Obstetrics and Gynecology, Cedars-Sinai Medical Center, Los Angeles, CA USA; 6 Psychology Department, University of Colorado – Colorado Springs, Colorado Springs, CO USA; 7 Department of Psychology, University of California – Los Angeles, Los Angeles, CA USA

**Keywords:** Pregnancy, GrimAgeAccel, Epigenetic age, Gestational length, Birthweight

## Abstract

**Background:**

Advanced biological aging, as measured by epigenetic aging indices, is associated with early mortality and morbidity. Associations between maternal epigenetic aging indices in pregnancy and pregnancy outcomes, namely gestational length and birthweight, have not been assessed. The purpose of this study was to examine whether epigenetic age during pregnancy was associated with gestational length and birthweight.

**Results:**

The sample consisted of 77 women from the Los Angeles, CA, area enrolled in the Healthy Babies Before Birth study. Whole blood samples for DNA methylation assay were obtained during the second trimester (15.6 ± 2.15 weeks gestation). Epigenetic age indices GrimAge acceleration (GrimAgeAccel), DNAm PAI-1, DNAm ADM, and DNAm cystatin C were calculated. Gestational length and birthweight were obtained from medical chart review. Covariates were maternal sociodemographic variables, gestational age at blood sample collection, and pre-pregnancy body mass index. In separate covariate-adjusted linear regression models, higher early second trimester GrimAgeAccel, *b(SE)* = − .171 (.056), *p* = .004; DNAm PAI-1, *b(SE)* = − 1.95 × 10^−4^ (8.5 × 10^−5^), *p* = .004; DNAm ADM, *b(SE)* = − .033 (.011), *p* = .003; and DNAm cystatin C, *b(SE)* = 2.10 × 10^−5^ (8.0 × 10^−5^), *p* = .012, were each associated with shorter gestational length. Higher GrimAgeAccel, *b(SE)* = − 75.2 (19.7), *p* < .001; DNAm PAI-1, *b(SE)* = − .079(.031), *p* = .013; DNAm ADM, *b(SE)* = − 13.8 (3.87), *p* = .001; and DNAm cystatin C, *b(SE)* = − .010 (.003), *p* = .001, were also associated with lower birthweight, independent of gestational length.

**Discussion:**

Higher maternal prenatal GrimAgeAccel, DNAm PAI-1, DNAm ADM, and DNAm cystatin C were associated with shorter gestational length and lower birthweight. These findings suggest that biological age, as measured by these epigenetic indices, could indicate risk for adverse pregnancy outcomes.

## Background

Adverse pregnancy outcomes, such as preterm birth (< 37 weeks gestation) and low birthweight (< 2500 g), affect approximately 10% and 8.3% of pregnancies in the USA, respectively [1, 2]. Adverse pregnancy outcomes are leading causes of neonatal death [3] and have long-term implications for child and fetal development, including poorer cognitive and physiological outcomes [4, 5]. Shorter gestation and lower birthweight are also costly on a societal level, for example, resulting in millions of dollars in healthcare costs, and loses in future educational attainment and earning potential [6, 7]. Understanding the factors associated with risk for adverse pregnancy outcomes is a research priority.

Epigenetic age indices are proposed to capture unique epigenetic signatures of biological aging. As an indicator of biological age, they are highly correlated with chronological age [8, 9] but are also associated with disease morbidity and death, e.g., cancer [10, 11] and cardiovascular disease [12], and early mortality [13–15], pointing to the utility of epigenetic age as a biomarker of biological aging [16]. In the pregnancy context, older maternal chronological age is associated with higher rates of adverse birth outcomes, including shortened gestational length and lower birthweight [17–21], suggesting that biological aging might be involved in risk. However minimal research has tested whether maternal biological aging, as opposed to chronological aging, is also a prognostic marker.

The few studies that exist suggest that prenatal markers of maternal biological age, e.g., telomere length, an indicator of cellular aging, could be associated with pregnancy outcomes. With respect to shorter gestational length, lower maternal peripheral blood telomerase activity and shortened leukocyte telomere length during pregnancy (key elements of biological aging) were associated with shorter gestational length [22, 23]. And with respect to lower birthweight, one study reported that shortened leukocyte telomere length was not associated with risk for having a small-for-gestational-age infant [24]. Although a few studies suggest that maternal variation in distinct methylation profiles are associated with gestational length [25–27], epigenetic estimates of maternal biological age as prognostic of birth outcomes have not been tested, although accelerated placenta epigenetic aging, as indexed by the Horvath clock, has been associated with lower birthweight [28]. Indeed, no studies to date have specifically considered whether maternal epigenetic biological age is associated with offspring birthweight. Given that epigenetic age acceleration is robustly associated with morbidity and mortality in non-pregnant adults, examining the association between epigenetic age and adverse pregnancy outcomes is an important research direction [29].

The purpose of this study is to test whether maternal epigenetic age acceleration during pregnancy is associated with major birth outcomes gestational length and birthweight. It was hypothesized that greater epigenetic age acceleration would be associated with shorter gestational length and lower birthweight. Exploratory analyses tested whether epigenetic age acceleration indices are more strongly associated with gestational length, independent of birthweight, or birthweight, independent of gestational length.

## Results

### Study characteristics

#### Sample characteristics

A sample of 77 women were recruited from Los Angeles, CA, into this pilot study as part of the Healthy Babies Before Birth (HB3) cohort, a longitudinal study designed to test the impact of antenatal maternal mood on pregnancy and postpartum outcomes. Demographics and previous pregnancy information were obtained at study entry and are presented in Table 1. The majority of participants were White (49%) or Latina (23%), married or cohabiting (87%), and primiparous (61%).

**Table 1:** Sample characteristics (*n* = 77)

Variable	Mean ± SD or % (*N*)	Range
Age (years)	33.1 ± 4.81	22.0–45.0
Marital status (married)	87% (65)	
Per Capita Household income ($1000)	52.5 ± 40.3	2.83–200
Education (years)	16.9 ± 2.90	12.0–26.0
Race/ethnicity		
White	49% (38)	
Black	10% (8)	
Latina	23% (18)	
Asian	12% (9)	
Multi-race	5% (4)	
Smoking (yes)	0% (0)	
Pre-pregnancy BMI	25.0 ± 5.82	16.8–36.4
Parity (primiparous)	61% (47)	
GA at first assessment (weeks)	15.6 ± 2.15	12.0–24.4
GA at birth (weeks)	39.4 ± 1.47	32.9–42.0
Birthweight (g)	3371 ± 507	1086–4624
Age acceleration residual	.135 ± 3.79	− 9.03–12.6
Senescence T cells	.643 ± 2.68	− 5.98–10.4
IEAA	.252 ± 3.63	− .973–13.3
EEAA	.702 ± 4.15	− 16.1–9.27
PEAA	32.5 ± 6.44	17.8–58.2
GrimAgeAccel	.500 ± 3.17	− 5.59–11.3
DNAm PAI-1	19.8 ± 2176	− 5578–5777
DNAm ADM	1.17 ± 14.1	− 34.6–43.8
DNAm B2M	8541 ± 77,648	− 138,633–326,766
DNAm cystatin C	2728 ± 20,284	− 32,366–74,727
DNAm GDF	4.41 ± 84.3	− 118–527
DNAm leptin	−55.2 ± 1584	− 3448–3296
DNAm TIMP1	135 ± 564	− 932–1632
DNAm smoking pack years	.076 ± 6.80	− 11.3–27.4
DNAm TL	− .008 ± .162	− .399–.424

*ADM* adrenomedullin, *B2M* β2 Microglobulin, *BMI* body mass index, *EEAA* extrinsic epigenetic age acceleration, *IEAA* intrinsic epigenetic age acceleration, *GA* gestational age, *GDF* growth and differentiation factor, *GrimAgeAccel* GrimAge acceleration, *PAI-1* plasminogen activation inhibitor-1, *PEAA* phenotypic epigenetic age acceleration, *TIMP-1* tissue inhibitor of metalloproteinases-1, *TL* telomere

#### Epigenetic age indices

Prenatal whole blood samples were collected at 15.6 ± 2.15 weeks gestation. DNA methylation was assayed using standardized protocols (see “Methods” section). Average epigenetic age index values are presented in Table 1. Fifteen epigenetic age indices were considered: age acceleration residual, age-adjusted senescent T cells, intrinsic epigenetic age acceleration (IEAA), extrinsic epigenetic age acceleration (EEAA), phenotypic epigenetic age acceleration (PEAA), GrimAge acceleration (GrimAgeAccel), and age-adjusted DNAm PAI-1, DNAm ADM, DNAm B2M, DNAm cystatin C, DNAm GDF, DNAm Leptin, DNAm TIMP-1, DNAm smoking pack years, and DNAm telomere (TL).

#### Pregnancy outcomes

Birthweight and gestational length were obtained by medical chart review. Average gestational length was 39.4 ± 1.47 weeks (range 32.9–42 weeks), with 4% (*N* = 3) of the sample meeting clinical guidelines for preterm birth (< 37 weeks gestation). Average birthweight was 3371 ± 507 g (range 1086–4624 g), with 1% (*N* = 1) meeting clinical guidelines for low birthweight (< 2500 g). Bivariate correlations are presented in Suppl Table 1.

### Pregnancy epigenetic age and gestational length

**Fig. 1:**
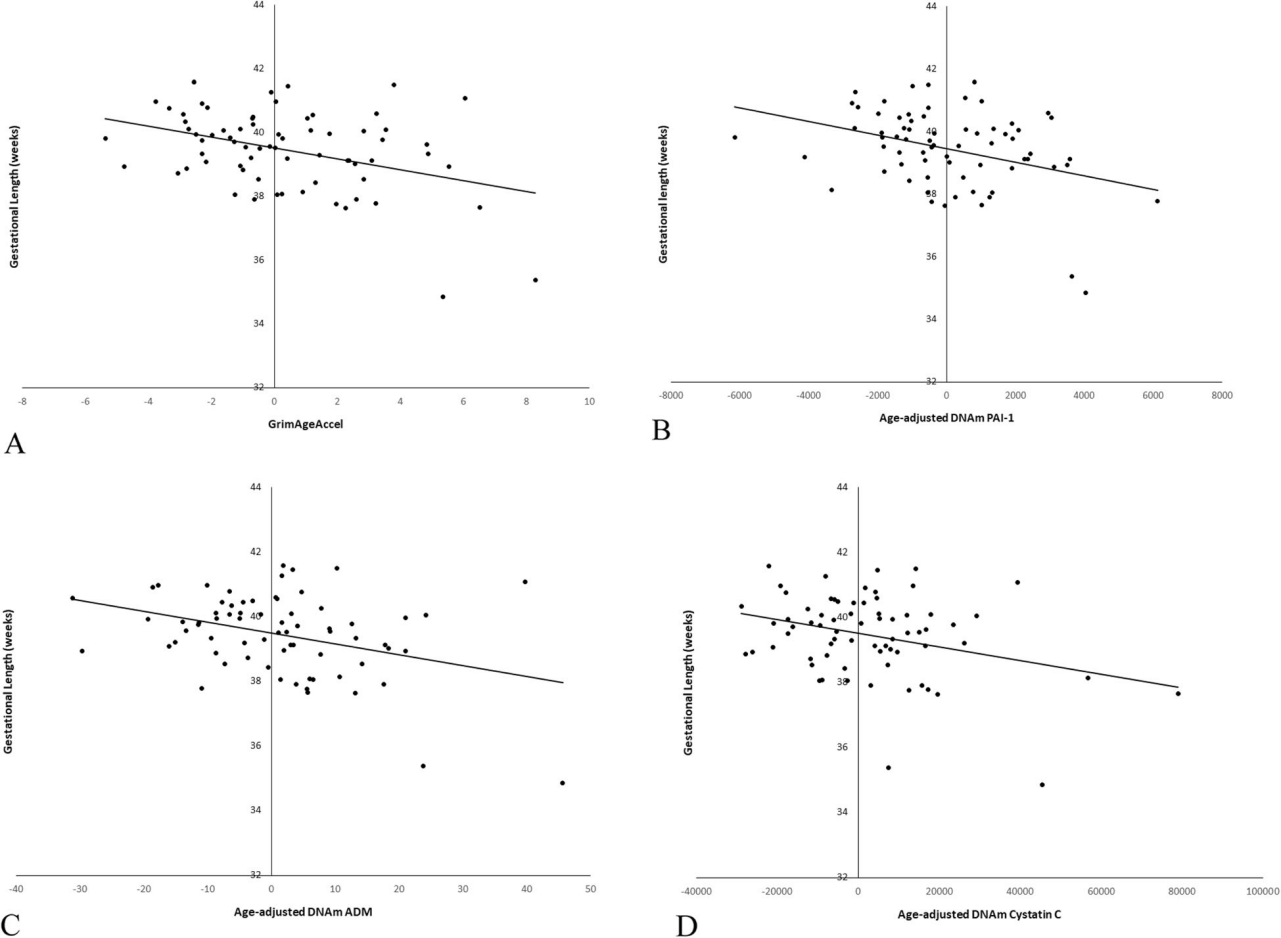
Associations between **a** GrimAgeAccel, **b** age-adjusted DNAm PAI-1, **c** age-adjusted DNAm ADM, and **d** age-adjusted DNAm cystatin C and gestational length, adjusting for covariates. Higher early second trimester GrimAgeAccel, DNAm PAI-1, DNAm ADM, and DNAm Cystatin C were associated with shorter gestational length

Results of linear regression models testing associations between gestational length and epigenetic age indices are presented in Table 2. Covariates were demographics, gestational age at blood sample collection, parity, and pre-pregnancy body mass index (BMI). Independent of covariates, four early second trimester epigenetic age indices were associated with gestational length. Higher GrimAgeAccel, an epigenetic age marker enriched for DNA methylation sites that are surrogate biomarkers for blood plasma proteins related to morbidity and mortality and cigarette smoking, estimated by packs per year [30], was associated with shorter gestational length, *b* = − .171, *SE* = .056, *p* = .004 (Fig. 1a). Higher DNAm PAI-1, *b* = − 1.95 × 10^−4^, *SE* = 8.5 × 10^−5^, *p* = .004 (Fig. 1b); DNAm ADM, *b* = − .033, *SE* = .011, *p* = .003 (Fig. 1c); and DNAm cystatin C, *b* = − 2.10 × 10^−5^, *SE* = 8.0 × 10^−5^, *p* = .012 (Fig. 1d), all DNA methylation indices validated by identifying the CpG sites most associated with blood plasma concentrations of plasminogen activator inhibitor (PAI)-1, adrenomedullin (ADM), and Cystatin C, respectively [30], were also associated with shorter gestational length. None of the other epigenetic age indices were associated with gestational length, *p*’s > .091.

**Table 2:** Summary of linear regression models testing associations between pregnancy outcomes, gestational length and birthweight, from epigenetic age indices, controlling for demographics, gestational age at blood sample collection, and pre-pregnancy BMI

Outcome	Predictor	*b*	*SE*	*β*	*p*
Gestational length	Age acceleration residual	− .041	.046	− .116	.384
	Senescent T cells	− .050	.069	− .093	.474
	IEAA	− .044	.046	− .123	.345
	EEAA	.017	.049	.045	.735
	PEAA	.005	.040	.018	.905
	**GrimAgeAccel**	− **.172**	**.053**	− **.398**	**.002**
	**DNAm PAI-1**	− **2.17 × 10**^**−4**^	**7.2 × 10**^**−5**^	− **.375**	**.004**
	**DNAm ADM**	− **.033**	**.011**	− **.353**	**.003**
	DNAm B2M	− 4.02 × 10^−6^	2.0 × 10^−5^	− .207	.091
	**DNAm cystatin C**	− **2.10 × 10**^−**5**^	**8.0 × 10**^−**5**^	− **.304**	**.012**
	DNAm GDF	− 1.69 × 10^4^	.002	− .011	.930
	DNAm leptin	− 6.24 × 10^−5^	1.1 × 10^−4^	− .071	.586
	DNAm smoking pack years	− .044	.028	− .200	.125
	DNAm TIMP1	3.71 × 10^−4^	3.1 × 10^−4^	− .150	.234
	DNAm TL	− .819	1.16	− .096	.482
Birthweight	Age acceleration residual	− 13.4	16.7	− .108	.428
	Senescent T cells	.695	25.0	.004	.978
	IEAA	− 15.6	16.7	− .124	.353
	EEAA	− 11.4	17.8	− .088	.524
	PEAA	− 12.6	14.3	− .134	.382
	**GrimAgeAccel**	− **71.9**	**18.5**	− **.471**	**< .001**
	**DNAm PAI-1**	− **.081**	**.026**	− **.397**	**.003**
	**DNAm ADM**	− **13.8**	**3.87**	− **.413**	**.001**
	DNAm B2M	− .002	.001	− .223	.075
	**DNAm Cystatin C**	− **.010**	**.003**	− **.403**	**.001**
	DNAm GDF	− .346	.691	− .063	.618
	DNAm leptin	.006	.041	.018	.894
	DNAm smoking pack years	− 17.8	10.2	− .228	.086
	DNAm TIMP1	− .151	.111	− .173	.179
	DNAm TL	217	420	.072	.608

All epigenetic age indices were adjusted for chronological age

*ADM* adrenomedullin, *B2M* β2 Microglobulin, *EEAA* extrinsic epigenetic age acceleration, *IEAA* intrinsic epigenetic age acceleration, *GDF* growth and differentiation factor, *GrimAgeAccel* GrimAge acceleration, *PAI-1* plasminogen activation inhibitor-1, *PEAA* phenotypic epigenetic age acceleration, *TIMP-1* tissue inhibitor of metalloproteinases-1, *TL* telomere

### Pregnancy epigenetic age and birthweight

**Fig. 2:**
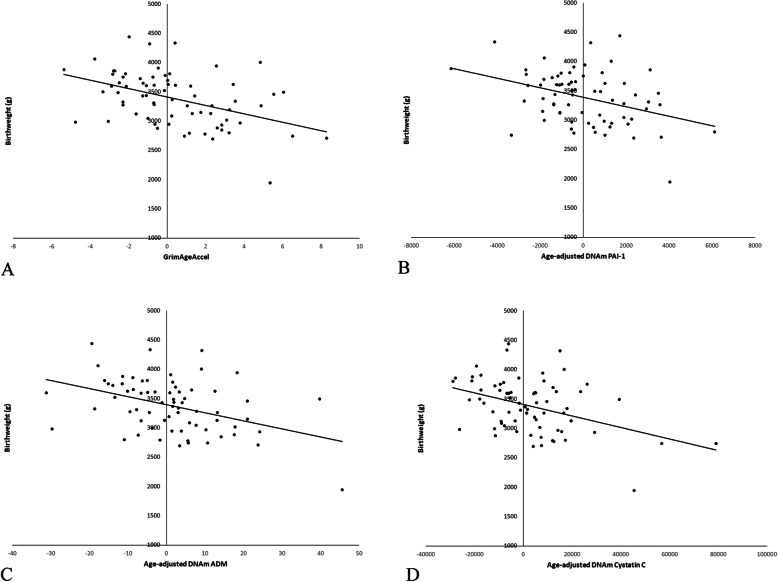
Associations between **a** GrimAgeAccel, **b** age-adjusted DNAm PAI-1, **c** age-adjusted DNAm ADM, and **d** age-adjusted DNAm cystatin C and birthweight, adjusting for covariates. Higher early second trimester GrimAgeAccel, DNAm PAI-1, DNAm ADM, and DNAm cystatin C were associated with lower birthweight

Results of linear regression models testing associations between birthweight and epigenetic age indices are presented in Table 2. Covariates were demographics, gestational age at blood sample collection, and pre-pregnancy BMI. Independent of covariates, higher early second trimester GrimAgeAccel, *b* = − 75.2, *SE* = 19.7, *p* < .001 (Fig. 2a); DNAm PAI-1, *b* = − .079, *SE* = .031, *p* = .013 (Fig. 2b); DNAm ADM, *b* = − 13.8, *SE* = 3.87, *p* = .001 (Fig. 2c); and DNAm cystatin C, *b* = − .010, *SE* = .003, *p* = .001 (Fig. 2d), were associated with lower birthweight. None of the other epigenetic age indices were associated with birthweight, *p*’s > .075.

### Exploratory analyses: birthweight and gestational length

Finally, linear regression models were run to determine if epigenetic age indices GrimAgeAccel, DNAm PAI-1, DNAm ADM, and DNAm cystatin C were associated with birthweight independent of gestational length, and gestational length independent of birthweight. Independent of gestational length, higher early second trimester GrimAgeAccel, *b* = − 44.9, *SE* = 18.1, *p* = .016; DNAm ADM, *b* = − 8.37, *SE* = 3.72, *p* = .028; and DNAm cystatin C, *b* = − .006, *SE* = .003, *p* = .019, continued to be associated with lower birthweight. However, GrimAgeAccel, *b* = − .080, *SE* = .053, *p* = .137; DNAm ADM, *b* = − .015, *SE* = .011, *p* = .159; and DNAm cystatin C, *b* = − 7.54 × 10^−6^, *SE* = 8.0 × 10^−5^, *p* = .341, were not associated with gestational length independent of birthweight. DNAm PAI-1 was also not associated with either gestational length independent of birthweight, *b* = − 1.11 × 10^−4^, *SE* = 6.90 × 10^−5^, *p* = .113, nor birthweight independent of gestational length, *b* = − .044, *SE* = .025, *p* = .077.

## Discussion

The purpose of this study was to determine if maternal epigenetic age acceleration indices during pregnancy were associated with gestational length and birthweight. Fifteen epigenetic age indices were considered: age acceleration residuals, IEAA, EEAA, PEAA, GrimAgeAccel, and age-adjusted DNAm PAI-1, DNAm ADM, DNAm B2M, DNAm cystatin C, DNAm GDF-15, DNAm leptin, DNAm TIMP-1, DNAm telomere, DNAm smoking pack years, and proportion of senescent T cells. Independent of covariates, higher early second trimester maternal GrimAgeAccel, DNAm PAI-1, DNAm ADM, and DNAm cystatin C were significantly associated with shorter gestational length; no other epigenetic clock estimate variables were significant. In addition, higher early second trimester maternal GrimAgeAccel, DNAm ADM, and DNAm cystatin C were significantly associated with having a lower birthweight baby, while other epigenetic estimates of biological age of the mother were not. In exploratory analyses, higher early second trimester maternal GrimAgeAccel, DNAm ADM, and DNAm cystatin C continued to significantly be associated with lower birthweight of the child independent of gestational length of the pregnancy, but maternal GrimAgeAccel, DNAm ADM, and DNAm cystatin C were no longer associated with gestational length when controlling for birthweight. These findings suggest that maternal epigenetic age acceleration indices, specifically GrimAgeAccel, DNAm ADM, and DNAm cystatin, are associated with risk for adverse pregnancy outcomes, and highlight the potential role of maternal biological aging in birth outcomes.

Contrary to hypotheses, only a subset of epigenetic age acceleration indices was associated with gestational length and birthweight, specifically GrimAgeAccel, DNAm PAI-1, DNAm ADM, and DNAm cystatin C. Several epigenetic aging indices have been developed over the last decade, with a trend toward moving from epigenetic indices that were predominately validated based on chronological age (e.g., Horvath epigenetic clock) toward indices that are validated or enriched for disease morbidity and biological risk factors. GrimAgeAccel is calculated by taking the residual of GrimAge regressed onto chronological age. GrimAge is constructed as a composite marker calculated from chronological age, sex, and epigenetic surrogate markers for 7 plasma proteins (adrenomedullin, β-2-microglobulin, cystatin-C, growth differentiation factor 15, leptin, plasminogen activator inhibitor 1, and tissue inhibitor metalloproteinase 1) and smoking pack years, based on self-reported smoking data [30], and is strongly associated with death. DNAm PAI-1, DNAm ADM, and DNAm cystatin C are the epigenetic surrogate marker for plasminogen activator inhibitor 1, adrenomedullin, and cystatin-C, respectively [30]. Plasminogen activator inhibitor-1 is a glycoprotein involved in suppressing fibrinolysis or the breakdown of blood clots, the production of which increases during pregnancy [31, 32]; adrenomedullin is a vasodilator peptide hormone, the production of which increases during pregnancy [33]; and cystatin-C is a protein that is typically used as an indicator of kidney function, the production of which decreases during pregnancy [34]. As only GrimAgeAccel, DNAm PAI-1, DNAm ADM, and DNAm cystatin C were significant markers of pregnancy outcomes, this suggests that these epigenetic age acceleration indices might be most sensitive in capturing unique biological processes not represented in the other epigenetic age acceleration indices, and which could be salient to physiological processes related to pregnancy outcomes, particularly birthweight. However, it is not clear what biological activity is captured by these indices. For example, in the validation study, associations between DNAm PAI-1, DNAm ADM, and DNAm cystatin C and their respective plasma biomarkers were moderate (*r*’s = .36–.39) [30], suggesting that these indices could be capturing additional biological activity. As such, additional well-powered research is warranted before firm conclusions should be drawn. Future research should begin to explore the unique biological activity or processes captured by the epigenetic age acceleration indices, and the role this could play in placenta development and function, with implications for intrauterine growth, and the biology of parturition (e.g., cervical integrity and rupturing of membranes), with implications for gestational length. Regardless, our findings suggest that early second trimester GrimAgeAccel, DNAm PAI-1, DNAm ADM, and DNAm cystatin C could be useful in markers of risk for shorter gestational length or low birthweight.

This is the first study to specifically consider associations between maternal epigenetic age acceleration indices during pregnancy and pregnancy outcomes. Of note, this study contributes to a burgeoning field of research that proposes biological aging of the mother and the placenta could contribute to timing of birth. Indeed, maternal chronological age is prognostic of birth outcomes [17–21], while less clear is the role of aging biology. Accelerated placenta epigenetic aging, as indexed by the Horvath clock, has been associated with lower birthweight [28]. In parallel, the placenta also can experience telomere length shortening that induces cellular senescence and the release of secretory factors (senescence-associated secretory phenotype) that promote parturition through increased inflammatory signaling cascades. In women with an older biological age, these processes may be accelerated [23, 35, 36]. As placental tissue was not evaluated here, additional research is needed to determine whether the placenta is responsible for associations between GrimAgeAccel and DNAm PAI-1 with risk for shorter gestation and lower birthweight.

Interestingly, findings from prior epigenome-wide association studies that reported associations between shorter gestation and differences in DNA methylation in immune and regulatory pathways is consistent with what is reported here, in that GrimAgeAccel and DNAm PAI-1 are epigenetic age acceleration indices developed based on immunological markers. A study of 154 women who presented with threatened preterm labor found differences in whole blood epigenetic profiles between women who did go on to have a preterm delivery and those women who did not, specifically in pathways related to signal transduction, transport, stress response, metabolic processes, immune system processes, mRNA processing, and cellular modification process [25]. Differences in DNA methylation patterns were also detected in a larger sample of 300 African American women [26]. Earlier birth was associated with hypomethylation at sites in the promoter regions of *CYTIP* and *LINC00114*, genes that are potentially involved in leukocyte trafficking, T-cell receptor signaling, transcriptional control, epigenetic modification, post-transcriptional control of mRNA, and cell differentiation [26]. In parallel, prior work has identified heightened circulating markers of inflammation, greater inflammatory gene expression, and related transcription factors [i.e., nuclear factor kappa B (NFкB), activator protein 1 (AP1)] among pregnant women who go on to have an early delivery and lower birthweight newborns [37, 38]. Collectively, this suggests that alterations in DNA methylation pathways that are shared with biological aging might also be related to immune and inflammatory activity that play a role in risk for shorter gestation. As biological aging is highly entwined with inflammatory processes [39], this potential pathway in the context of adverse birth outcomes warrants further research.

Associations between gestational length and birthweight and DNAm ADM and DNAm cystatin C are also consistent with previous literature. Both DNAm ADM and DNAm cystatin C were validated from markers involved in hemodynamics, including vasodilation and kidney glomerular filtration rate, respectively [31, 32, 34]. Hemodynamic adaptations are essential during pregnancy to ensure adequate circulation to the placenta and to meet the increased metabolic demands of the developing fetus, and include remodeling of cardiac tissue, increases in blood volume, increased cardiac output, and vasodilation of the vasculature and kidneys [40, 41]. Higher levels of cystatin C are associated with risk for preeclampsia diagnosis [42–45], a cardio-metabolic disease of pregnancy driven by distress signals released by an ischemic placenta. Preeclampsia is also associated with risk for shorter gestational length and low birthweight [46]. Similarly, higher levels of adrenomedullin could be associated with increased risk for low birthweight and shorter gestational length [47]. It is possible that DNAm ADM and DNAm cystatin C are indicators of these hemodynamic processes. However, these mechanisms must be interpreted with caution. Associations between DNAm ADM and DNAm cystatin C with protein serum levels of these markers are modest, *r* = .38–.39 [30], and these indices are calculated from blood samples. Nonetheless, these findings suggest that age-adjusted DNAm ADM and DNAm cystatin C could be capturing biological activity relevant to pregnancy hemodynamics and are potentially indicative of increased risk for shorter gestational length and lower birthweight.

Of interest, DNAm TL was not associated with gestational length or birthweight. This is contrary to previous research reporting associations between shorter telomere length in leukocytes and shorter gestational length [22, 23] but is consistent with a reported lack of association between leukocyte telomere length and birthweight [24]. It is possible that DNAm TL and leukocyte telomere length capture different physiological processes, accounting for differences in the pattern of findings. DNAm smoking pack years was also not associated with pregnancy outcomes, despite associations between maternal smoking or exposure to second-hand smoke and risk for shorter gestational length and lower birth weight [48–50]. DNAm smoking pack years was validated based on self-reported smoking habits. None of the women here reported any cigarette use during pregnancy, and few (> 10%) reported any tobacco use in the 3 months prior to conception. It is possible that no association was detected because there were too few smokers or previous smokers in the sample. Additional research is needed to better understand DNAm TL and DNAm smoking pack years and how they relate to leukocyte telomere length and self-reported smoking, respectively, in the context of pregnancy.

There are several study limitations to consider. Although our sample of *N* = 77 was larger and more diverse than other studies that have examined associations between pregnancy outcomes and DNA methylation patterns, it is still relatively small for DNA methylation research. Our sample has also generally higher income and educated, and generally low risk with few cases of clinically classified preterm birth or low birthweight. Future research should replicate these analyses in a larger, more diverse sample with high risk women. Finally, epigenetic age indices have not been validated in pregnant women. This is an important gap given that pregnancy is characterized by unique and dynamic physiological adaptations across physiological systems, including the immune, cardio-metabolic, and neuroendocrine [51]. How these adaptations affect blood cell DNA methylation patterns, or whether these patterns are stable or shift over the course of pregnancy, is not known. Future research should validate epigenetic clocks in pregnant samples.

## Conclusions

In sum, to the best of our knowledge, this is the first study to assess whether maternal epigenetic age acceleration indices during pregnancy are associated with gestational length and birthweight. Higher maternal GrimAgeAccel, DNAm PAI-1, DNAm ADM, and DNAm cystatin C during the early second trimester were associated with shorter gestation and lower birthweight, independent of maternal demographics, pre-pregnancy BMI, parity, and gestational age at blood sampling. Maternal biological aging, as indexed by GrimAgeAccel, DNAm PAI-1, DNAm ADM, and DNAm cystatin C, during pregnancy could affect risk for adverse pregnancy outcomes.

## Methods

### Participants

A sample of 77 women was recruited into this pilot study as part of the Healthy Babies Before Birth (HB3) cohort. HB3 study inclusion criteria were 18 years of age or older and singleton pregnancies up to 12 weeks gestation at time of recruitment. Exclusion criteria were medical intake involving current substance abuse, HIV-positive status, current smoking, or medications that could affect inflammatory processes, e.g., glucocorticoids. The current sample focused on women recruited at one of the two study sites (Los Angeles, CA), who had whole blood samples collected at study entry in early pregnancy. Study data were collected and managed using REDCap electronic data capture tool [52].

### Protocol

Demographics and previous pregnancy information were obtained at study entry. Whole blood samples were collected at the first (8–16 weeks gestation) or second (20–26 weeks) pregnancy visit. Birthweight and gestational length were obtained by medical chart review.

### DNA methylation

DNA was extracted from whole blood and assayed for DNA methylation by the UCLA Neurosciences Genomics Core using the Illumina Infinium HumanMethylation450 BeadChip (Illumina, Inc., San Diego, CA; 485,577 CpG sites). DNA methylation data were pre-processed as per standard protocols [9, 53], with detailed methods previously reported [54].

### Epigenetic age indices

The epigenetic age of each blood sample was estimated using previously published methods [54] and using algorithms available through an online DNA methylation calculator [9] (https://labs.genetics.ucla.edu/horvath/dnamage/).

*DNA methylation age* (DNAm age; years), or biological age, was calculated using the Horvath method [9]. Differences between chronological and biological age are captured by the *age acceleration residual*, such that positive values indicate accelerated biological aging. *Age-adjusted proportions of exhausted or senescent CD8+ T cells* (CD8 + CD28-CD45FA- T cells) were calculated using estimation procedures validated by Horvath and described elsewhere [9, 55], adjusting for chronological age. Higher proportion of age-adjusted senescent CD8+ T cells is indicative of immunosenescence [56, 57]. *Intrinsic epigenetic age acceleration (IEAA)* captures the intrinsic biological age of immune cells, independent of age-related changes in immune cell populations in the blood. *Extrinsic epigenetic age acceleration (EEAA)* captures immune cell biological aging due to both intrinsic immune cell age and age-driven changes in immune cell populations. *Phenotypic epigenetic age acceleration (PEAA)* is calculated in accordance with the Levine method, using sites that were selected based on associations with both chronological age and phenotypic indicators of aging [58]. *GrimAgeAccel* is an epigenetic age marker enriched for DNA methylation sites that are surrogate biomarkers for blood plasma proteins related to morbidity and mortality and cigarette smoking, estimated by packs per year [30]. *DNAm PAI-1*, *DNAm ADM*, *DNAm*, *B2M*, *DNAm cystatin C*, *DNAm GDF*, *DNAm leptin*, *DNAm TIMP1*, and *DNAm smoking pack years* are surrogate DNA methylation index validated by identifying the CpG sites most associated with blood plasma protein concentrations or self-reported smoking [30].

### Covariates

Demographics [race/ethnicity (White or not White), years of education, per capita household income, marital status (married and/or cohabiting or not)]; gestational age at blood sample collection; parity (nulliparous vs. parous^1^); and pre-pregnancy BMI were included as covariates. Demographics were collected at study entry. Gestational age at blood sample collection was calculated by subtracting conception date (determined through ultrasound) from the assessment date. At study entry, participants reported their last pre-pregnancy weight. Height was measured at baseline by study personnel using a balance-beam scale. Pre-pregnancy BMI (kg/m^2^) was calculated by taking last pre-pregnancy weight (kg) and dividing by height squared (m^2^). Smoking prior to and during pregnancy was also considered as a covariate. Less than 10% (*N* = 6) reported nicotine use prior to pregnancy. Given that this variable was highly skewed, and preconception nicotine use was not correlated with epigenetic age indices, *p*’s > .129, or birth outcomes, *p*’s > .691, preconception nicotine use was not included as a covariate in analyses. No women reported any cigarette use during pregnancy.

### Analytic strategy

All analyses were run using SPSS v. 24 [59]. Data was checked for outliers and normality prior to analysis. One birthweight outlier was Winsorized to − 3 SD (1850 g), and two gestational length outliers were Winsorized to − 3 SD (34.6 weeks).^2^ Separate linear regression models were fit testing associations between gestational length and birthweight and each epigenetic age indices, controlling for covariates (demographics, gestational age at assessment, parity, and pre-pregnancy BMI).

## Supplementary information

**Additional file 1.** Supplemental Table 1. Correlations

## Data Availability

Data are available upon request from J. Carroll and C. Dunkel Schetter.

## Abbreviations

ADM: Adrenomedullin
AP1: Activator protein 1
B2M: β2 Microglobulin
BMI: Body mass index
DNAm: DNA methylation
EEAA: Extrinsic epigenetic age acceleration
HB3: Healthy Babies Before Birth
IEAA: Intrinsic epigenetic age acceleration
GDF: Growth and differentiation factor
GrimAgeAccel: GrimAge acceleration
NFкB: Nuclear factor kappa B
PAI-1: Plasminogen activator inhibitor-1
PEAA: Phenotypic epigenetic age acceleration
TIMP-1: Tissue inhibitor of metalloproteinases-1
TL: Telomere

## Footnotes

1 Pattern of results does not change whether parity is treated as a dichotomous or continuous variable.

2 Pattern of results is the same regardless of whether outliers are Winsorized or quartile linear regression is used.
