# Supplementary material for: Epigenetic age and pregnancy outcomes: GrimAge acceleration is associated with shorter gestational length and lower birthweight
Source: Clin Epigenetics. 2020 Aug 6;12:120. doi: 10.1186/s13148-020-00909-2 (PMC7409637; doi:10.1186/s13148-020-00909-2)
Supplement: Supplementary file 1 — Additional file 1. Supplemental Table 1. Correlations [file 13148_2020_909_MOESM1_ESM.docx]

Supplemental Table 1. Correlations

| Variable | 1. | 2. | 3. | 4. | 5. | 6. | 7. | 8. | 9. | 10. | 11. | 12. | 13. | 14. | 15. |
| --- | --- | --- | --- | --- | --- | --- | --- | --- | --- | --- | --- | --- | --- | --- | --- |
| 1. AgeAccelRes |  |  |  |  |  |  |  |  |  |  |  |  |  |  |  |
| 1. Sen T cells | -0.215 |  |  |  |  |  |  |  |  |  |  |  |  |  |  |
| 1. IEAA | **0.969** | -0.161 |  |  |  |  |  |  |  |  |  |  |  |  |  |
| 1. EEAA | **0.433** | 0.119 | **0.419** |  |  |  |  |  |  |  |  |  |  |  |  |
| 1. PEAA | **0.397** | **0.248** | **0.428** | **0.410** |  |  |  |  |  |  |  |  |  |  |  |
| 1. GrimAgeAccel | **0.307** | 0.189 | **0.345** | **0.234** | **0.435** |  |  |  |  |  |  |  |  |  |  |
| 1. DNAm PAI-1 | 0.103 | **0.263** | 0.155 | -0.008 | **0.363** | **0.562** |  |  |  |  |  |  |  |  |  |
| 1. Gestational length | -0.035 | **-0.259** | -0.056 | -0.049 | -0.193 | **-0.362** | **-0.285** |  |  |  |  |  |  |  |  |
| 1. Birthweight | -0.100 | -0.134 | -0.115 | 0.022 | -0.122 | **-0.313** | -0.165 | **0.558** |  |  |  |  |  |  |  |
| 1. Race/ethnicity | -0.224 | 0.162 | **-0.231** | -0.025 | 0.037 | 0.095 | 0.076 | **-0.302** | -0.042 |  |  |  |  |  |  |
| 1. Per capita household income | -0.145 | -0.061 | -0.118 | -0.164 | -0.049 | -0.108 | -0.127 | 0.005 | -0.062 | -0.128 |  |  |  |  |  |
| 1. Education | 0.037 | **-0.230** | 0.058 | -0.155 | 0.011 | -0.141 | -0.159 | 0.152 | -0.120 | -0.174 | **0.386** |  |  |  |  |
| 1. Marital status | -0.092 | -0.162 | -0.076 | -0.056 | **-0.243** | -0.218 | -0.076 | 0.148 | 0.079 | -0.073 | 0.172 | 0.163 |  |  |  |
| 1. Parity | **0.278** | -0.031 | 0.217 | 0.070 | 0.167 | -0.079 | -0.083 | 0.012 | 0.046 | -0.064 | **-0.358** | 0.046 |  |  |  |
| 1. GA at assessment | 0.166 | 0.006 | 0.160 | 0.114 | **0.250** | **0.301** | **0.302** | -0.030 | 0.195 | -0.077 | -0.014 | -0.103 | 0.062 | 0.075 |  |
| 1. Pre-pregnancy BMI | -0.064 | 0.138 | 0.023 | 0.011 | 0.198 | 0.210 | **0.336** | 0.015 | 0.197 | 0.053 | **-0.306** | -0.211 | -0.081 | -0.070 | 0.096 |

Note: GA = gestational age. **Bold** = significant at p < .05
